# *CD44* Methylation Levels in Androgen-Deprived Prostate Cancer: A Putative Epigenetic Modulator of Tumor Progression

**DOI:** 10.3390/ijms26062516

**Published:** 2025-03-11

**Authors:** Virginia Valentini, Raffaella Santi, Valentina Silvestri, Calogero Saieva, Giandomenico Roviello, Andrea Amorosi, Eva Compérat, Laura Ottini, Gabriella Nesi

**Affiliations:** 1 Department of Molecular Medicine, Sapienza University of Rome, 00161 Rome, Italy; virginia.valentini@uniroma1.it (V.V.); valentina.silvestri@uniroma1.it (V.S.); laura.ottini@uniroma1.it (L.O.); 2 Department of Health Sciences, Section of Anatomic Pathology, University of Florence, 50139 Florence, Italy; raffaella.santi@unifi.it; 3 Risk Factors and Lifestyle Epidemiology Unit, Institute for Cancer Research and Clinical Network (ISPRO), 50134 Florence, Italy; c.saieva@ispro.toscana.it; 4 Department of Health Sciences, Section of Clinical Pharmacology and Oncology, University of Florence, 50139 Florence, Italy; giandomenico.roviello@unifi.it; 5 Department of Health Sciences, “Magna Graecia” University of Catanzaro, 88100 Catanzaro, Italy; amorosi@unicz.it; 6 Department of Pathology, Medical University of Vienna, 1090 Vienna, Austria; eva.comperat@muv.ac.at

**Keywords:** prostate cancer, androgen deprivation, epigenetics, *CD44*

## Abstract

Epigenetic changes have been reported to promote the development and progression of prostate cancer (PCa). Compared to normal prostate tissue, tumor samples from patients treated with androgen-deprivation therapy (ADT) show the hypermethylation of genes primarily implicated in PCa progression. A series of 90 radical prostatectomies was retrospectively analyzed. A total of 46 patients had undergone surgery alone (non-treated) and 44 had received ADT prior to surgery (treated). Promoter methylation analysis of the candidate genes possibly involved in PCa response to ADT (*AR*, *ESR1*, *ESR2*, *APC*, *BCL2*, *CD44*, *CDH1*, *RASSF1*, *ZEB1*) was conducted by pyrosequencing. The mRNA expression of differentially methylated genes was investigated by quantitative real-time PCR. Intratumoral microvessel density and ERG expression were also assessed using immunohistochemistry. A statistically significant difference in *CD44* promoter methylation levels was found, with higher levels in the non-treated cases, which accordingly showed lower *CD44* gene expression than the treated cases. Moreover, lower *ESR1* methylation levels were associated with higher ERG expression, and the *CD44* methylation levels were increased in ERG-overexpressing tumors, particularly in the treated cases. Our data suggest an interplay between ERG expression and the epigenetic modifications in key genes of prostate tumorigenesis, and that *CD44* promoter methylation could serve as a promising molecular biomarker of PCa progression under androgen-deprived conditions.

## 1. Introduction

Prostate cancer (PCa) is the most common malignancy and the second leading cause of cancer death among men in the United States and in Western countries [1]. The androgen receptor (AR) signaling pathway is essential for the physiologic development and maintenance of the prostate gland [2]. At initial diagnosis, the great majority of PCa are androgen dependent. Androgen-deprivation therapy (ADT) is the gold standard treatment for advanced PCa and has rapid beneficial effects in men with metastatic PCa; however, most patients eventually experience disease progression (castration-resistant prostate carcinoma, CRPC) [3]. A large number of studies have investigated the molecular mechanisms of PCa hormonal resistance, attributing a central role to alterations that cause persistent canonical AR signaling [4]. Nevertheless, other mechanisms (e.g., AR-independent bypass pathways, tumor stem cells) have also been advocated to explain progression to androgen independence [4,5,6,7].

Angiogenesis is vital for tumor development and progression; its clinical and prognostic significance has been widely investigated in many cancer types including PCa. Several studies have suggested an inverse correlation between intratumoral microvessel density (MVD) and recurrence-free survival in PCa patients, and it has been shown that MVD can predict biochemical relapses after a radical prostatectomy [8].

*ERG* (an *ETS*-related gene), a member of the *ETS* transcription factor family, is essential for various cellular processes, including proliferation, differentiation, apoptosis, tissue remodeling, and angiogenesis. ERG overexpression is one of the key factors in transforming localized, aggressive cancer into metastatic cancer. In a complex network of transcriptional crosstalk among ERG, AR, and epigenetic programming, ERG has been proven to attenuate androgen-regulated transcription in PCa [9].

It has been postulated that genetic and epigenetic changes, notably DNA methylation, can contribute to PCa initiation and progression [10]. Aberrant promoter methylation of the genes involved in hormonal response, tumor cell invasion/metastasis, cell cycle control, and DNA damage repair have been described as ultimately leading to altered gene expression in PCa [11,12]. Over the past few years, there has been increasing interest in epigenetic markers for PCa diagnosis and prognosis [13,14,15,16], and a promoter methylation analysis of the genes implicated in prostate tumorigenesis and consistently hypermethylated in PCa has been proposed as a specific and sensitive method for cancer detection and molecular characterization [14,17].

Progression to CRPC is associated with numerous epigenetic alterations that promote the survival of neoplastic cells at metastatic sites [18]. Hypermethylation of the genes primarily involved in tumor progression may enable the determination of ADT-treated patients at risk of developing CRPC [19]. Lately, various DNA methylation signatures of metastatic CRPC capable of predicting patient prognosis have been identified in tissue and liquid biopsy samples [20].

In this study, therapy-related differences were elucidated by comparing the methylation profiles and phenotypic features, such as ERG status and MVD, of ADT-treated and non-treated PCa cases. We analyzed the promoter methylation levels of the candidate genes possibly implicated in the response to ADT (i.e., *AR*, *ESR1*, *ESR2*, *APC*, *BCL2*, *CD44*, *CDH1*, *RASSF1*, *ZEB1*), for which aberrant DNA methylation has been correlated with modified gene expression [21,22]. In particular, CD44 shows promise as a potential biomarker of progression and resistance to therapy in various cancers. In PCa, a high expression of CD44 is linked to cancer stem cell-like properties, tumor aggressiveness, recurrence, and poor prognosis [23,24]. Its dysregulation occurs through several mechanisms including epigenetic changes [24]; therefore, understanding these changes may provide valuable insights into the impact of ADT on tumor biology and therapeutic response.

## 2. Results

### 2.1. Clinicopathologic Characteristics of PCa Cases

As shown in Table 1, the age of onset ranged from 49 to 73 years, with a mean age of 65.3 (SD = ±5.4). With regard to the Gleason Score (GS) of the non-treated patients, 27 (58.7%) were diagnosed as grade group 1 or 2, while 19 (41.3%) were classified as grade group 3, 4, or 5. Concerning the tumor stage (pT), 47 (52.2%) patients presented pT2 and 43 (47.8%) pT3 disease. The majority had negative surgical margins (n = 80, 88.9%) and no lymph node involvement (n = 68, 90.7%). Follow-up data were available for 86 patients, with an overall mean observation of 56.2 months (±31.1, range 6–112 months). In the survival analysis, 67 of the 86 patients were disease-free, with no significant difference between the treated (28 out of 41, 68.3%) and non-treated cases (39 out of 45, 86.7%) (*p* = 0.067). No statistical significance emerged for the clinicopathologic variables analyzed.

### 2.2. Intratumoral Microvessel Density (MVD) Related to Hormone Treatment

The PCa cases were divided into two groups according to the MVD median value (31.5)—45 with lower values and 45 with higher values (Table S1). The mean (±DS) and median MVD in the specimens from the non-treated patients (n = 46, 51.1%) were 33.3 (±6.6) and 31.4, respectively, whereas they were 31.7 (±6.6) and 31.8 in treated patients (n = 44, 47.7%). The intratumoral MVD did not differ in the two groups (*p* = 0.83), and no significant association was found between the MVD groups and the other clinicopathologic characteristics.

### 2.3. ERG Immunohistochemical Expression Related to Hormone Treatment

**Figure 1:**
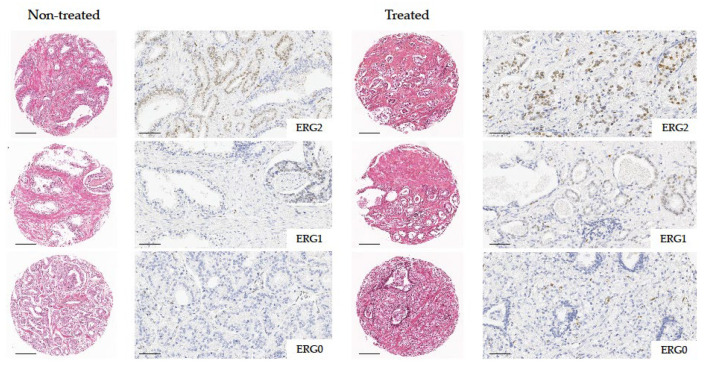
Representative ERG immunostaining on TMA samples from treated and non-treated PCa cases (ERG0, negative staining; ERG1, positive staining in <50% of neoplastic cells; ERG2, positive staining in ≥50% of neoplastic cells). Magnification 5× and 20× (scale bars 250 μm and 100 μm, respectively).

Overall, ERG immunoreactivity was detected in 29 (32.2%) PCa specimens. Of these, 11 (12.2%) exhibited positive staining in <50% of the neoplastic cells (ERG1) and 18 (20.0%) in ≥50% (ERG2). A total of 61 (67.8%) PCa samples did not express ERG following immunohistochemistry (ERG0) (Figure 1). The ERG expression did not differ in the two treatment groups (*p* = 0.33), and no correlation was observed between the ERG immunoreactivity and the selected clinicopathologic parameters (Table S2).

### 2.4. Methylation Analysis Related to Hormone Treatment

**Figure 2:**
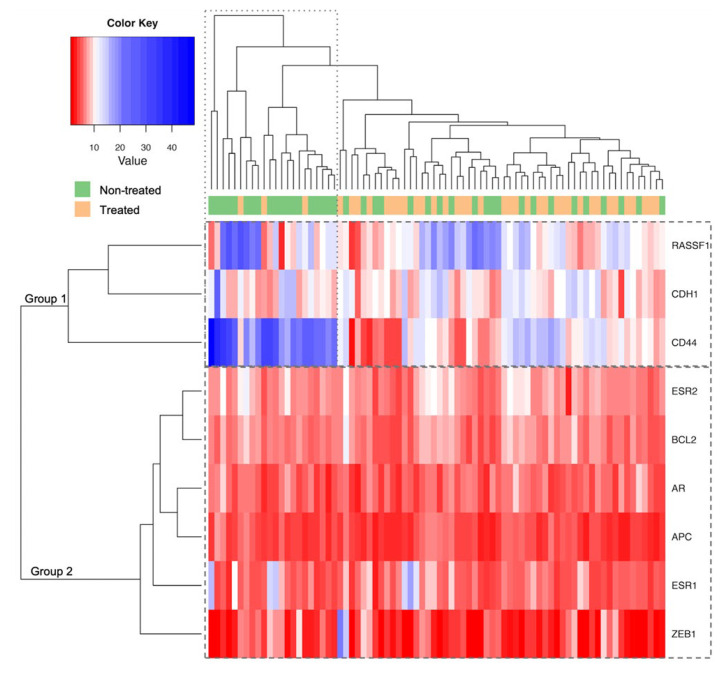
Unsupervised hierarchical clustering analysis of promoter methylation levels of 9 genes in 78 PCa cases.

Using a gene-specific approach, the promoter methylation levels of the *AR*, *ESR1*, *ESR2*, *APC*, *BCL2*, *CD44*, *CDH1*, *RASSF1*, and *ZEB1* genes were measured in 87 PCa cases, comprising 42 treated and 45 non-treated patients. To evaluate the methylation profile, an unsupervised hierarchical clustering analysis was performed on 78 cases (38 treated and 40 non-treated), for which the promoter methylation levels of all the genes examined were available. As outlined in Figure 2, two main groups of clustered genes were identified, namely group 1 (i.e., *RASSF1*, *CDH1*, and *CD44*) showing higher promoter methylation levels and group 2 (i.e., *ESR2*, *BCL2*, *AR*, *APC*, *ESR1*, and *ZEB1*) displaying lower promoter methylation levels. No clear distinctive clusters emerged between the treated and non-treated cases; however, higher *CD44* methylation levels were mainly observed in the non-treated cases.

**Figure 3:**
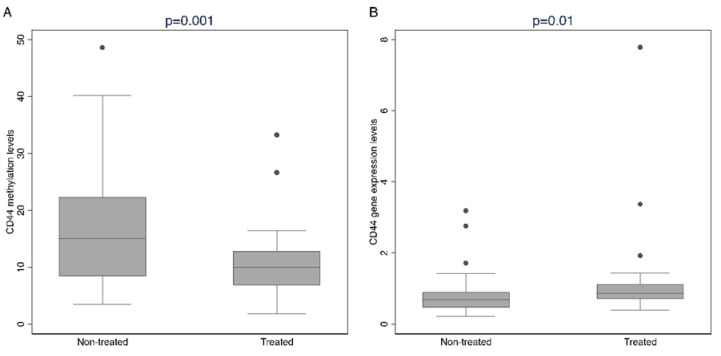
(**A**) *CD44* promoter methylation levels (expressed as median %). (**B**) *CD44* gene expression levels (expressed as 2^−ΔCt^) in treated and non-treated PCa cases.

Indeed, a comparison of the median methylation levels revealed a significant difference for *CD44* (*p* = 0.001), where the non-treated cases had higher *CD44* promoter methylation levels than the treated cases, with median levels of 15.20% and 10.00%, respectively (Table 2, Figure 3A). In accordance with the higher methylation levels observed in the non-treated cases, the *CD44* gene expression levels were significantly lower in the non-treated group (*p* = 0.01; Figure 3B), suggesting that the *CD44* DNA methylation levels may be inversely correlated with the mRNA levels (Figure S1). Following immunohistochemistry, the neoplastic cells were found to express CD44 in 40/90 (44.4%) cases. Of these, 25/90 (27.8%) showed positive staining in <50% of the PCa cells (CD44-1) and 15/90 (16.7%) in ≥50% (CD44-2) (Figure S2).

No statistical differences in the promoter methylation levels were observed for the other genes investigated.

### 2.5. CD44 Methylation Levels According to MVD and ERG Expression

When the gene promoter median methylation levels (%) were examined for their distribution according to the MVD (low vs. high), no relevant differences were found (Table S3). Contrary to this, a statistically significant difference was seen for *ESR1* in relation to ERG immunoreactivity (*p* = 0.014; Table 3), with lower methylation levels being associated with higher ERG expression. Additionally, the *CD44* methylation levels varied in the three groups identified on the basis of ERG expression (*p* = 0.004; Table 3). Given the disparity of the *CD44* promoter methylation levels between the treated and non-treated cases, their relationship with ERG expression was explored (Table 4). The *CD44* promoter methylation levels differed significantly in the treated patients relative to the ERG status (*p* = 0.029) but not in the untreated patients (*p* = 0.11).

## 3. Discussion

ADT is the primary therapeutic option for locally advanced and metastatic PCa. However, after an initial regression, the cancer cells become castration resistant, rendering tumor relapse inevitable [4]. In view of the paucity of information on the risk factors that affect CRPC development, clarification of the phenotypic and molecular properties of ADT-treated PCa may provide insights into the mechanisms of disease progression [25].

In this study, a total of 90 PCa cases—46 undergoing surgery alone (non-treated) and 44 receiving 3-month neoadjuvant ADT prior to radical prostatectomy (treated)—were analyzed and compared. No significant differences in the main clinicopathologic characteristics, MVD, or ERG immunoreactivity emerged between the non-treated and hormonally treated cases. Therefore, the epigenetic signatures were examined to ascertain whether they could identify the distinct molecular subtypes of PCa. Using pyrosequencing, we tested the promoter methylation status of the genes involved in the hormonal and cancer progression pathways known to be pivotal in prostate tumorigenesis. A statistically significant difference was seen for *CD44*, with higher promoter methylation levels in the non-treated cases. Since altered promoter methylation has been associated with aberrantly transcriptional expression of the corresponding gene [26], we evaluated *CD44* gene expression and found that the non-treated PCa cases with higher *CD44* methylation levels displayed lower *CD44* gene expression than the treated cases. A significant correlation has previously been observed between *CD44* hypermethylation and the higher stages of PCa, indicating an important role for *CD44* methylation in PCa progression and metastasis [24]. Here, we provide the first evidence that the methylation status of *CD44* may be modulated under androgen-deprived conditions and could possibly serve as a marker of ADT response.

*CD44* encodes for a transmembrane glycoprotein involved in cell adhesion, cell–extracellular matrix interaction, and intracellular signaling transduction [27], and it is fundamental in the promotion of epithelial–mesenchymal transition (EMT)-related events [28]. EMT plays crucial roles in the metastatic process whereby epithelial cells lose cell-to-cell contact and polarity, increasing their migratory and invasive capabilities. In addition, CD44 overexpression has been reported to contribute to the proliferation, migration, invasion, and metastasis of PCa cells [29,30]. Interestingly, CD44 has also been identified as a cancer stem cell (CSC) marker in PCa; CD44-positive cells are more proliferative, clonogenic, tumorigenic, and metastatic than isogenic CD44-negative cells [30,31,32].

There is accumulated evidence that ADT may result in the expansion of CSCs [33,34]. Several authors claim that CRPC exhibits many phenotypes similar to CSCs, implying that a clonal expansion of the prostate CSC population may be responsible for PCa relapse and metastasis [35].

ADT causes tumor regression by inducing the apoptosis of the tumor epithelial luminal and stromal cells that express AR. This may lead to a decrease in luminal cells, along with an increase in basal intermediate prostate CSCs and AR-negative PCa cells, which exhibit a high proliferation index and repopulate the tumor through transit to the luminal layer, thus giving rise to CRPC [36].

The gene expression of CSC surface markers, such as *CD44*, has been shown to be regulated at the transcriptional level by DNA methylation in PCa [37]. Our data add evidence for the possible expansion of a CSC population following ADT and for its role in driving castration resistance, although functional studies using marker-based or marker-free methods for CSC enrichment and analysis are mandatory to validate this hypothesis [38].

Considering the relationship between epigenetic regulation, including DNA methylation and ERG expression in PCa [39,40], we further assessed the gene promoter methylation levels regarding ERG immunostaining. Lower methylation levels in the promoter of the *ESR1* gene, which codes for the estrogen receptor (ER)-α, were found to correlate with strong ERG reactivity, implying a synergistic effect of the two proteins. ERG is overexpressed in approximately half of prostatic adenocarcinomas as a result of a gene fusion with the androgen-driven promoter of the *TMPRSS2* gene [41]. In the case of AR inactivity, *TMPRSS2-ERG* can be under the control of other androgen-independent mechanisms such as ERα [42], while *TMPRSS2-ERG* fusions are associated with a distinct genetic signature consistent with ER signaling [9].

We also observed that *CD44* promoter methylation levels differed significantly in relation to ERG status in the treated but not in the untreated cases, suggesting that hormone therapy may have a different impact on specific PCa subtypes. It has previously been reported that ERG overexpression leads to a strong induction of adhesive mesenchymal-like genes (e.g., *CD24* and *CD44*), which reflects an ERG-driven pattern of malignancy [40]. Of note, ERG has been found to enhance the susceptibility of neoplastic cells to high-dose androgen therapy [43]. Our data, showing higher promoter methylation and lower expression of *CD44* in the treated cases overexpressing ERG, point to an interaction between ERG and the epigenetic changes in the key genes under androgen-deprived conditions.

This study has the following limitations: the small number of patients involved; its retrospective nature; the lack of clinical information such as preoperative risk-stratification variables; and the determination of ERG status limited to immunohistochemical detection of the protein without an analysis of the genomic alterations. Moreover, this is a pilot study and the cause-and-effect relationship between *CD44* methylation and ADT still needs to be investigated in order to validate the clinical significance of *CD44* methylation. Finally, the mechanisms leading to *CD44* hypermethylation, including the dysregulation of DNA methyltransferases (DNMTs), have not been thoroughly addressed. Indeed, experimental evidence points to a key role for DNMTs in the development of aggressive PCa phenotypes such as CRPC, and distinct gene expression profiles of DNMTs have been found at different timings of PCa progression [44].

In conclusion, our results provide insights into the characterization of ADT-treated PCa, suggesting an interplay between epigenetic modifications and ERG overexpression. Additionally, *CD44* promoter methylation shows potential as a molecular biomarker of PCa progression under androgen-deprived conditions, and it is worthy of further verification in a large-scale cohort.

## 4. Materials and Methods

### 4.1. Tissue Specimens

A retrospective series of 90 radical prostatectomies was analyzed at the Division of Anatomic Pathology, University of Florence, Florence, Italy. A total of 46 cases (51.1%) had undergone surgery alone (non-treated) and 44 (48.9%) underwent surgery preceded by ADT (treated), consisting of luteinizing hormone-releasing hormone (LH-RH) agonists administered by subcutaneous injection for 3 months. The serum CgA levels were preoperatively measured in 78 cases (39 treated and 39 non-treated) by means of a two-sided “sandwich” technique, with two selected antibodies binding to different epitopes of human CgA (Epitope Diagnostics, Inc.–EDI Human Chromogranin A ELISA Kit, San Diego, CA, USA; normal range 0–120 ng/mL).

Hematoxylin and eosin (H&E)-stained slides of the tumors were reviewed, and pathologic staging was performed in accordance with the 2017 AJCC TNM classification [45]. The GS was assessed in the non-treated cases, and these were stratified according to the WHO grade group defined as follows: 1 (GS ≤ 6); 2 (GS 3 + 4 = 7); 3 (GS 4 + 3 = 7); 4 (GS 4 + 4 = 8/5 + 3 = 8/3 + 5 = 8), and 5 (GS 9–10) [46]. In the treated cases, the GS was not evaluated, since the grading of prostatic tissue following hormonal therapy is considered misrepresentative of the actual disease and may lead to inaccurate prognostication [47].

Cases were selected on the availability of archival formalin-fixed, paraffin-embedded (FFPE) tissue specimens. The study design was approved by the local ethics committee (Prot. 16806_bio) and the procedures adhered to the tenets of the Declaration of Helsinki.

### 4.2. Traditional Whole Sections and Tissue Microarray Construction

The traditional whole sections comprised consecutive 4 µm thick sections cut from each donor block. Representative areas of tumor tissue were selected from these blocks, based on H&E staining, and three cores (0.6 cm in diameter) were transferred from each donor block to the recipient block. Serial 3 µm sections were cut from the resulting tissue microarray (TMA) blocks and stained with H&E to verify the presence of tumor tissue in the cores. A total of 270 tumor tissue cores were examined, and a surface area of 0.848 mm^2^ was analyzed for each case.

### 4.3. Immunohistochemical Analysis

Immunostaining was achieved using the BenchMark^®^ ULTRA automated staining system (Ventana Medical Systems, Tucson, AZ, USA). For antigen retrieval, the slides were heated with Cell Conditioning Solution 1 (CC1). The sections were incubated with mouse monoclonal anti-CD31 (clone JC70, Cell Marque, Rocklin, CA, USA), rabbit monoclonal anti-ERG (clone EPR3864, Ventana, Tucson, AZ, USA), and rabbit monoclonal anti-CD44 (clone SP37, Ventana). Negative controls were simultaneously performed by substituting the primary antibody with a Ventana dispenser filled with non-immune serum.

The MVD was measured by examining the traditional whole sections stained for CD31 at a low magnification (×40, ×100) to identify the most vascularized area of the tumor (the so-called hotspot). Within these hotspots, 5 fields at ×400 magnification were studied and the vessels were counted, applying the criteria based on those of Weidner et al. [48]. The average count of all the fields (MVD mean) was calculated and recorded. For the statistical analysis, the median was set as the cut-off point to distinguish high and low values of MVD.

ERG immunostaining was appraised on the TMA sections and considered to be positive if its expression was nuclear. Only cases with positive normal endothelial staining (internal control) were scored. Tumor tissue samples were divided into the following three groups: ERG0 when the neoplastic cells did not express ERG; ERG1 when positive staining was observed in <50% of the neoplastic cells, regardless of intensity; ERG2 when positive staining was observed in ≥50% of the neoplastic cells, regardless of intensity.

The CD44 expression was evaluated on whole PCa tissue sections. Only those cells exhibiting a strong membranous staining pattern were considered positive. Immunoreactivity was semi-quantitatively assessed, and the tumor samples were subdivided into the following three groups: CD44-0 when the neoplastic cells did not express CD44; CD44-1 when positive staining was observed in <50% of the neoplastic cells; CD44-2, when positive staining was observed in ≥50% of the neoplastic cells.

### 4.4. Methylation Analysis

For each sample, DNA was extracted from 10 µm thick FFPE tumor sections using the EpiTect Plus FFPE Lysis Kit (Qiagen, Hilden, Germany), following the manufacturer’s instructions. DNA was isolated from microdissected tumor samples consisting of at least 60–70% neoplastic cells.

The promoter methylation of the *AR*, *ESR1*, *ESR2*, *APC*, *BCL2*, *CD44*, *CDH1*, *RASSF1*, and *ZEB1* genes was assessed in 87 PCa cases, with 3 excluded due to poor DNA quality. DNA bisulfite modification was carried out with the EpiTect Plus DNA Bisulfite Kit (Qiagen), following the manufacturer’s instructions, which ensured the complete conversion and minimal degradation of the treated DNA. Methylation analysis was conducted on a total of 39 CpG sites by pyrosequencing, a highly sensitive and reproducible method that provides absolute quantitative information from each site analyzed. Pyrosequencing was performed with Pyromark Q24 (Qiagen) according to a previously described protocol [49].

A commercial assay, including primers for amplification and sequencing (PyroMark CpG assay, Qiagen), was used for each gene (Table S4). In each assay, controls of bisulfite conversion were included in the dispensation order. Data analysis was accomplished with the PyroMark Q24 System (Qiagen), determining the degree of methylation at each CpG position, in a sequence through the C/T ratio. Target CpGs were estimated by converting the resulting pyrograms to numerical values for the peak heights. For each gene, the methylation levels were expressed as the median of methylation percentage at all the CpG sites investigated. Higher and lower promoter methylation levels were based on the cut-off point of 10%.

### 4.5. CD44 Gene Expression Analysis

For each sample, the total RNA was extracted from 10 µm FFPE tissue sections using the miRNeasy FFPE Kit (Qiagen), following the manufacturer’s protocol. All samples were microdissected to enrich the tumor tissue. The RNA quantity and quality were assessed with the Agilent 2100 Bioanalyzer (Agilent Technologies, Santa Clara, CA, USA). *CD44* gene expression analysis was performed on 84 PCa samples, with 6 excluded due to poor RNA quality. Reverse transcription was carried out using the High-Capacity cDNA Reverse Transcription Kit (Life Technologies, Carlsbad, CA, USA), according to the manufacturer’s instructions, and qRT-PCR was conducted on a 7500 Fast Real-Time PCR platform (Applied Biosystem, Carlsbad, CA, USA). Pre-designed TaqMan assays for *CD44* and housekeeping genes (RPL37A and GUSB) were employed (Life Technologies, Carlsbad, CA, USA), with the latter being used to normalize gene expression. Each sample was run in triplicate; relative gene expression levels were determined by the ΔCt method (where ΔCt = Ct target gene − Ct mean of the housekeeping genes) and expressed as 2^–ΔCt^.

### 4.6. Statistical Analysis

To evaluate the association between ADT and the clinicopathologic characteristics of PCa patients (including intratumoral MVD and ERG expression in neoplastic cells), the chi-squared, Mann–Whitney, and Kruskal–Wallis tests were used as appropriate. Unsupervised hierarchical clustering was performed to analyze relevant clusters and co-methylation. Dendrograms and heatmaps were created by means of the Euclidean distance matrix and complete linkage. The non-parametric Mann–Whitney test was applied to compare the DNA methylation and gene expression values for each gene in the treated and non-treated tumor samples. A *p* value < 0.05 was considered as statistically significant. All statistical analyses were performed using R software (version 4.4.1).

## Figures and Tables

**Table 1:** Clinicopathologic characteristics of PCa patients according to androgen-deprivation therapy (ADT).

Characteristic ^a^	All Cases (n = 90; 100.0%)	Non-Treated Cases (n = 46; 51.1%)	Treated Cases (n = 44; 48.9%)	*p*-Value ^b^
Age at diagnosis				
Mean ± SD (range)	65.3 ± 5.4 (49–73)	65.3 ± 5.6 (49–73)	65.3 ± 5.3 (53–73)	0.89
Gleason grade group				
1–2	27 (58.7)	27 (58.7)	-	
3–5	19 (41.3)	19 (41.3)	-	
Tumor extent (pT)				
pT2	47 (52.2)	26 (56.5)	21 (47.7)	0.53
pT3	43 (47.8)	20 (43.5)	23 (52.3)	
Lymph node involvement (pN)				
pN0	68 (90.7)	33 (94.3)	35 (87.5)	0.44
pN1	7 (9.3)	2 (5.7)	5 (12.5)	
Surgical margins				
Negative	80 (88.9)	38 (82.6)	42 (95.5)	0.09
Positive	10 (11.1)	8 (17.4)	2 (4.5)	
Serum CgA, ng/mL				
Mean ± SD (range)	85.6 ± 55.2 (27.7–430)	88.2 ± 43.0 (32.6–218)	83.1 ± 65.8 (27.7–430)	0.29
Status				
No evidence of disease (NED)	67 (77.9)	39 (86.7)	28 (68.3)	0.067
Disease progression	19 (22.1)	6 (13.3)	13 (31.7)	

^a^ Some data are missing; ^b^ *p*-value from Mann–Whitney or chi-squared test, as appropriate; CgA, Chromogranin A.

**Table 2:** Gene promoter median methylation levels (%) in treated and non-treated PCa cases.

Gene	All Cases Median (n)	Non-Treated Cases Median (n)	Treated Cases Median (n)	*p*-Value ^a^
*AR*	5.00 (82)	5.00 (41)	5.00 (41)	0.59
*ESR1*	4.75 (81)	4.63 (40)	4.75 (41)	0.82
*ESR2*	6.40 (82)	6.60 (41)	6.30 (41)	0.34
*APC*	3.70 (80)	3.70 (39)	3.70 (41)	0.22
*BCL2*	6.00 (82)	6.00 (41)	5.70 (41)	0.57
*CD44*	11.70 (82)	15.20 (41)	10.00 (41)	**0.001**
*CDH1*	10.00 (81)	9.05 (40)	10.70 (41)	0.20
*RASSF1*	11.13 (80)	12.00 (39)	10.25 (41)	0.24
*ZEB1*	3.00 (76)	3.00 (38)	2.65 (38)	0.39

^a^ *p*-value from Mann–Whitney test. *p*-value < 0.05 (in bold) considered statistically significant.

**Table 3:** Gene promoter median methylation levels (%) according to ERG immunohistochemical expression *.

Gene	ERG0 (n = 61)	ERG1 (n = 11)	ERG2 (n = 18)	*p*-Value ^a^
*AR*	5.0 (55)	5.3 (9)	4.9 (18)	0.53
*ESR1*	5.0 (54)	4.5 (9)	4.3 (18)	**0.014**
*ESR2*	6.4 (55)	6.6 (9)	6.0 (18)	0.69
*APC*	3.7 (53)	3.7 (9)	3.4 (18)	0.80
*BCL2*	6.0 (55)	6.0 (9)	5.5 (18)	0.57
*CD44*	10.0 (55)	19.0 (9)	13.3 (18)	**0.004**
*CDH1*	9.8 (55)	11.3 (8)	9.8 (18)	0.72
*RASSF1*	11.5 (53)	12.3 (9)	10.0 (18)	0.50
*ZEB1*	3.0 (50)	2.7 (9)	2.7 (17)	0.83

* Percentage of positive staining cells (0: 0%; 1: <50%; 2: ≥50%); ^a^ *p*-value from Kruskal–Wallis test; *p*-values in bold denote statistical significance.

**Table 4:** Association between *CD44* methylation levels (%) and ERG expression * in treated and non-treated PCa cases.

	Non-Treated Cases (n = 46)		Treated Cases (n = 44)	
	% ^a^	*p*-Value ^b^	% ^a^	*p*-Value ^b^
ERG0	13.3	0.11	9.0	**0.029**
ERG1	21.8		15.4	
ERG2	15.5		10.7	

* percentage of positive staining cells (0: 0%; 1: <50%; 2: >50%); ^a^ median methylation levels of *CD44*; ^b^ *p*-value from Mann-Whitney or Kruskal-Wallis test as appropriate; *p*-values in bold denote statistical significance.

## Data Availability

The original contributions presented in the study are included in the article/Supplementary Materials, further inquiries can be directed to the corresponding author.

## Supplementary Materials

The following supporting information can be downloaded at: https://www.mdpi.com/article/10.3390/ijms26062516/s1.
