# Supplementary material for: CD44 Methylation Levels in Androgen-Deprived Prostate Cancer: A Putative Epigenetic Modulator of Tumor Progression
Source: Int J Mol Sci. 2025 Mar 11;26(6):2516. doi: 10.3390/ijms26062516 (PMC11942495; doi:10.3390/ijms26062516)

Table S1. Clinicopathologic characteristics of PCa patients according to intratumoral microvessel density (MVD)[a].

**Characteristic**[b]	**Low MVD (n=45) n (%)**	**High MVD (n=45) n (%)**	***p*-value**[c]
**ADT**			
Non-treated	24 (52.2)	22 (47.8)	0.83
Treated	21 (47.7)	23 (52.3)	
**Age at diagnosis (mean ± DS)**	65.5 ± 5.8	65.1 ± 5.0	0.39
**Gleason grade group**			
1-2	19 (54.3)	16 (45.7)	0.73
3-5	5 (45.5)	6 (54.5)	
**Tumor extent (pT)**			
pT2	22 (46.8)	25 (53.2)	0.67
pT3	23 (53.5)	20 (46.5)	
**Lymph node involvement (pN)**			
pN0	36 (52.9)	32 (47.1)	0.70
pN1	3 (42.9)	4 (57.1)	
**Surgical margins**			
Negative	39 (48.2)	42 (51.8)	0.49
Positive	6 (66.7)	3 (33.3)	
**Status**			
No evidence of disease (NED)	34 (50.7)	33 (49.3)	1.0
Disease progression	9 (47.4)	10 (52.6)	

[a]cases subdivided into two groups according to MVD median value; [b]some data are missing; [c]*p*-value from Mann-Whitney test or chi-square test as appropriate; ADT: androgen-deprivation therapy.

Table S2. Clinicopathologic characteristics of PCa patients according to ERG immunohistochemical expression*.

**Characteristic**[a]	**ERG0 (n=61) n (%)**	**ERG1 (n=11) n (%)**	**ERG2 (n=18) n (%)**	***p*-value**[b]
**ADT**				
Non-treated	29 (63.0)	5 (10.9)	12 (26.1)	0.33
Treated	32 (72.8)	6 (13.6)	6 (13.6)	
**Age at diagnosis (mean ± DS)**	65.4 ± 5.0	66.8 ± 5.9	64.1 ± 6.3	0.32
**Gleason grade group**				
1-2	22 (62.9)	5 (14.3)	8 (22.8)	0.34
3-5	7 (63.6)	0 (-)	4 (36.4)	
**Tumor extent (pT)**				
pT2	34 (72.3)	3 (6.4)	10 (21.3)	0.21
pT3	27 (62.8)	8 (18.6)	8 (18.6)	
**Lymph node involvement (pN)**				
pN0	44 (64.6)	8 (11.8)	16 (23.6)	0.35
pN1	6 (85.7)	1 (14.3)	0 (-)	
**Surgical margins**				
Negative	56 (70.0)	8 (10.0)	16 (20.0)	0.18
Positive	5 (50.0)	3 (30.0)	2 (20.0)	
**Status**				
No evidence of disease (NED)	49 (73.1)	5 (7.5)	13 (19.4)	0.065
Disease progression	10 (52.6)	5 (26.3)	4 (21.1)	

*tumor cell staining percentage (0: 0%; 1: <50%; 2: >50%); [a]some data are missing; [b]*p*-value from Kruskal-Wallis test or chi-square test as appropriate; ADT: androgen-deprivation therapy.

Table S3. Gene-promoter median methylation levels (%) according to intratumoral microvessel density (MVD)[a].

Gene	Low MVD (n=45)	High MVD (n=45)	*p*-value [b]
AR	4.70 (41)	5.20 (41)	0.22
ESR1	4.63 (40)	4.75 (41)	0.81
ESR2	6.40 (41)	6.60 (41)	0.42
APC	3.50 (40)	3.70 (40)	0.56
BCL2	5.70 (41)	6.00 (41)	0.27
CD44	10.40 (41)	12.40 (41)	0.58
CDH1	9.30 (40)	11.00 (41)	0.56
RASSF1	10.63 (40)	11.50 (40)	0.90
ZEB1	3.00 (39)	2.30 (37)	0.23

[a]cases subdivided into two groups by MVD median value; [b]*p*-value from Mann-Whitney test.

Table S4. Commercially available PyroMark CpG assays used for each gene analyzed.

Gene Symbol	GeneGlobe Id	Assay Name	N. of CpG sites included	Sequence to analyze
AR	PM00134106	Hs_AR_04_PM	3	YGTTAGGGTATTATATATTAGGTGYGGTGAAGTYGT
ESR1	PM00024612	Hs_ESR1_01_PM	4	YGATAGTTGYGGYGGYGGGT
ESR2	PM00166145	Hs_ESR2_05_PM	5	ATATTTGYGTYGTTAGATTGGGGTYGGGTTTTTTYGYGT
APC	PM00116809	Hs_APC_01_PM	6	GAYGGYGGGYGTYGYGATTTTTATTTATTGYGT
BCL2	PM00071295	Hs_BCL2_01_PM	3	TTTTTTTTTTTTTTTTTTGAATGAATYGTGTGAYGTTAYGT
CD44	PM00045598	Hs_CD44_01_PM	5	YGTYGYGGGGTTAGYGGGAGAAGAAAGTTAGTGYGT
CDH1	PM00171941	Hs_CDH1_01_PM	5	YGGTAGYGYGTTTTTATTTTTGTTTAGGAYGYGGT
RASSF1	PM00013300	Hs_RASSF1_02_PM	4	AGGGTYGTTGTTYGTTAGGTTTYGTAGGTTYGA
ZEB1	PM00043281	Hs_ZEB1_02_PM	3	GTGAGGGTYGGGTGYGGATGGGGAAGTGAGATAAGTATYGT

Figure S1. Scatter plot showing the inverse correlation between *CD44* promoter methylation levels (%) and gene expression levels ($2^{-\Delta Ct}$).

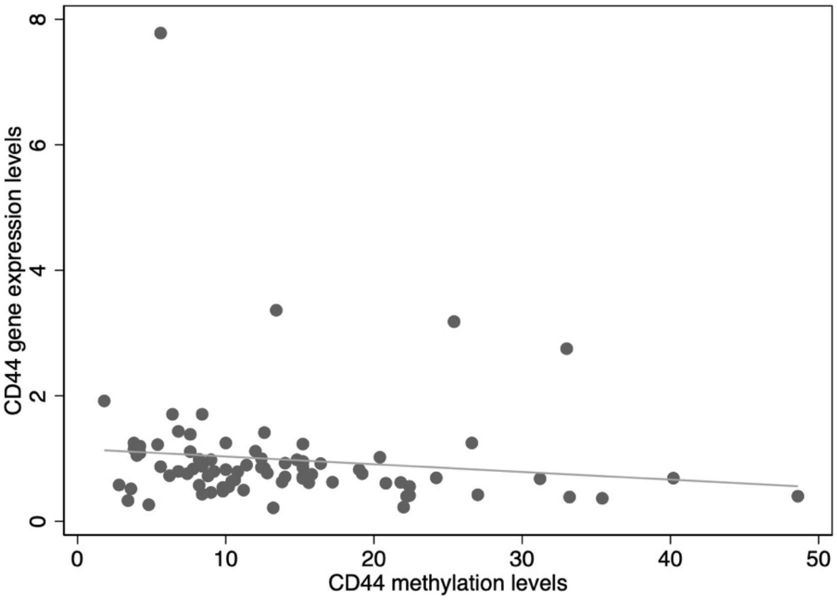

Figure S2. CD44 immunohistochemical expression of neoplastic cells (strong, continuous—*left* vs absent—*right,* scale bar 100μm).

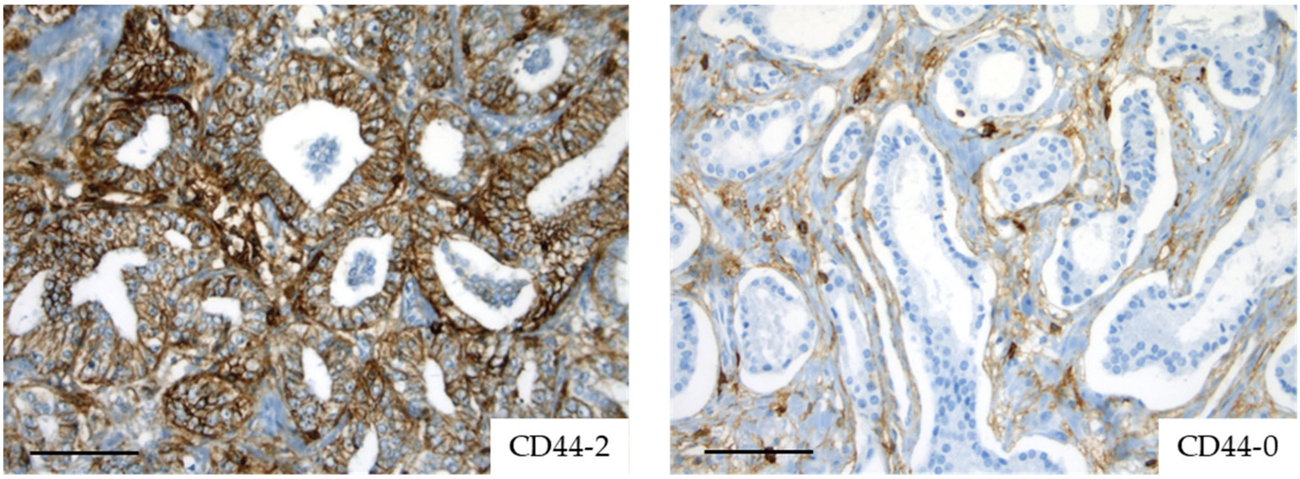

Supplement: Supplementary file 1 [file ijms-26-02516-s001.zip › ijms-3502991-supplementary.pdf]
